# Analysis of Interacting Proteins of Aluminum Toxicity Response Factor ALS3 and CAD in *Citrus*

**DOI:** 10.3390/ijms20194846

**Published:** 2019-09-29

**Authors:** Yan-Mei Wu, Yan-Yu Wang, Yang-Fei Zhou, Xin Meng, Zeng-Rong Huang, Li-Song Chen, Lin-Tong Yang

**Affiliations:** 1 Institute of Plant Nutritional Physiology and Molecular Biology, College of Resources and Environment, Fujian Agriculture and Forestry University, Fuzhou 350002, China; yanm898749741@163.com (Y.-M.W.); 15659395592@163.com (Y.-Y.W.); zyf15705989979@163.com (Y.-F.Z.); mengxin@kingenta.com (X.M.); hzrapaul@126.com (Z.-R.H.); lisongchen2002@hotmail.com (L.-S.C.); 2 Kingenta Ecological Engineering Group Co., Ltd, Linyi 276700, China; 3 Fujian Provincial Key Laboratory of Soil Environmental Health and Regulation, Fujian Agriculture and Forestry University, Fuzhou 350002, China

**Keywords:** *Citrus*, aluminum toxicity, ALS3, CAD, Y2H, BiFC

## Abstract

Aluminum (Al) treatment significantly decreased the dry weight (DW) of stem, shoot and whole plant of both *Citrus sinensis* and *C. grandis*, but did not change that of root. Al significantly decreased leaf DW of *C. grandis*, increased the ratio of root to shoot and the lignin content in roots of both species. The higher content of Al in leaves and stems and lignin in roots of *C. grandis* than that of *C. sinensis* might be due to the over-expression of Al sensitive 3 (ALS3) and cinnamyl alcohol deaminase (CAD) in roots of *C. grandis*, respectively. By using yeast-two-hybridazation (Y2H) and bimolecular fluorescence complementation (BiFC) techniques, we obtained the results that glutathione *S*-transferase (GST), vacuolar-type proton ATPase (V-ATPase), aquaporin PIP2 (PIP2), ubiquitin carboxyl-terminal hydrolase 13 (UCT13), putative dicyanin blue copper protein (DCBC) and uncharacterized protein 2 (UP2) were interacted with ALS3 and GST, V-ATPase, Al sensitive 3 (ALS3), cytochrome P450 (CP450), PIP2, uncharacterized protein 1 (UP1) and UP2 were interacted with CAD. Annotation analysis revealed that these proteins were involved in detoxification, cellular transport, post-transcriptional modification and oxidation-reduction homeostasis or lignin biosynthesis in plants. Real-time quantitative PCR (RT-qPCR) analysis further revealed that the higher gene expression levels of most of these interacting proteins in *C. grandis* roots than that in *C. sinensis* ones were consistent with the higher contents of lignin in *C. grandis* roots and Al absorbed by *C. grandis*. In conclusion, our study identified some key interacting components of Al responsive proteins ALS3 and CAD, which could further help us to understand the molecular mechanism of Al tolerance in citrus plants and provide new information to the selection and breeding of tolerant cultivars, which are cultivated in acidic areas.

## 1. Introduction

Aluminum (Al) is one of the most abundant elements in the earth’s crust, and is also the main element of soil inorganic minerals. Al exist as the form of Al(OH)^2+^ and Al^3+^ when the medium pH is < 5.0 [1]. When the soil is acidified, Al can be released from the solid phase as the form of Al^3+^ cation, which increases the activity of Al in the soil and has toxic effects on plants [2]. Although Al is not an essential element for plants, low doses of Al can promote the growth of a few species of plants such as tea and oak, but for most plants, even micro-molar (10 μM), Al can rapidly inhibit the growth of roots [3].

The most obvious feature of Al toxicity to plants is that Al inhibits cell elongation and cell division of root tips. It is generally believed that root tip is the main part of plant response to Al stress [4]. Research showed that Al toxicity caused a significant decrease in cell wall viscosity and elastic elongation, changed the physical and chemical properties such as pectin methylation, disorders distribution of galacturonic acid in root tip cell wall [3,5], and inhibited the root tip cell elongation and cell division, etc. [6].

Based on previous proteomic studies, Al sensitive 3 (ALS3) is expressed primarily in the root cortex, the leaf hydathodes and the phloem throughout the plant, and is involved in the redistribution of Al ions in plant cells [7,8]. Larsen et al. reported that the *At*ALS3 in *Arabidopsis* was responsible for the redistribution of Al ions and transport of Al ions to the shoots to protect the root tip from Al toxicity [9]. It has been proven that *Os*ALS1 can feedback-regulate the transport protein *Os*Nrat1, which can absorb Al cation, so that the Al cation is transported from cell wall to leaf bubble in root cell [10]. Cinnamyl alcohol deaminase (CAD) is a key enzyme in the biosynthesis of lignin in plant. Studies have shown that CAD is closely related to plant growth and response to biotic and abiotic stresses [11]. Zhou et al. used isobaric tags for relative and absolute quantification (iTRAQ) technology to analyze the apical proteomics of sorghum Al-sensitive cultivar BR007 and Al-resistant cultivar SC566, indicating that CAD4 protein was up-regulated in cultivar SC566 [12]. Our recent proteomic study also found that the protein abundance of CAD4 in *C. grandis* roots was significantly up-regulated by Al under low B level, which may resulted in the higher content of lignin in roots of *C. grandis* [13]. It is well known that proteins rarely achieve their biological functions in the form of independent individuals. The reaction network and transcriptional regulatory network between protein and protein interactions are important for the properly performing their regular function [14]. Yeast-two-hybridazation (Y2H) is a technique that can identify candidate interacting proteins of interesting protein; the candidate interacting proteins were further confirmed by the bimolecular fluorescence complementation (BiFC) technique. Recently, Y2H and BiFC have been widely used in the study of protein interaction in plants [15,16].

Citrus is one of the most important economic fruit trees worldwide. Most of the citrus orchards locate tropical and subtropical areas, where the soil is acidic or slightly acidic. According to our previous survey of 319 citrus orchards in Southern China, soil acidification is a challenge problem in these orchards, as the soil pH values of 90% of which were less than five [17]. Al could affect the normal growth and development of citrus, and finally reduce the quality and yield of citrus fruits [17,18]. Our previous studies have investigated the physiological, transcriptomic and proteomic profile of two citrus cultivars with different Al tolerance and some details of Al tolerance in citrus plants have been obtained [19,20,21]. However, the regulatory network and context of two Al responsive proteins ALS3 and CAD were still obscure. In order to unveil the molecular mechanism underpinning Al tolerance of citrus plants, Y2H, BiFC, real-time quantitative PCR (RT-qPCR) and other physiological methods were used to analysis the interacting proteins of ALS3 and CAD in response to Al. Several interacting proteins of ALS3 and CAD were successfully identified, which could help us to understand the regulatory mechanism of these two key proteins in Al tolerance in citrus.

## 2. Results

### 2.1. Effects of Al Treatment on the Plant DW and Root/Shoot in C. sinensis and C. grandis

**Figure 1:**
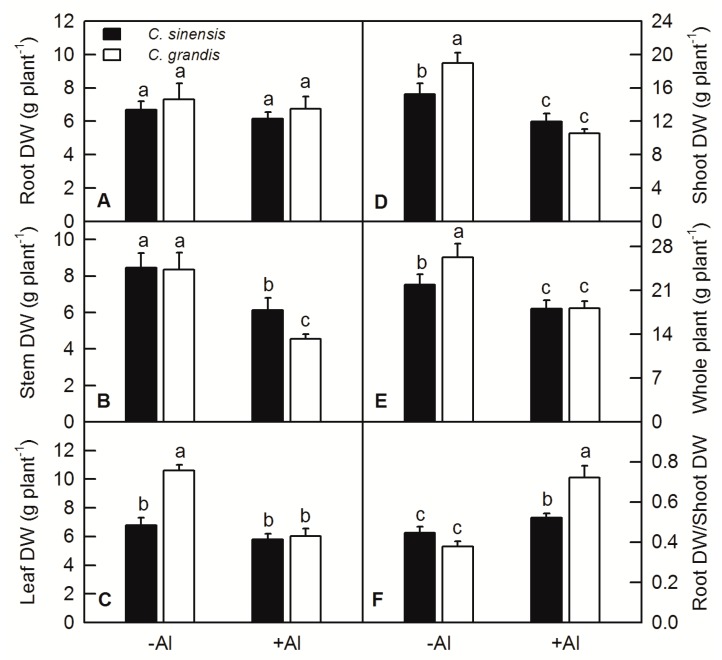
Effects of Al toxicity on dry weight (DW) of root (**A**), stem (**B**), leaf (**C**), shoot (stem + leaf) (**D**) and whole plant (**E**), and the ratio of root to shoot (**F**) in *C. sinensis* and *C. grandis*. Bars represent means ± SD (*n* = 10). Different letters above the bars indicate a significant difference at *p* < 0.05.

Al treatment significantly decreased the dry weight (DW) of stem, shoot and whole plant of both *C. sinensis* and *C. grandis* (Figure 1B,D,E), but did not change that of root (Figure 1A). Al treatment significantly decreased leaf DW of *C. grandis* and did not change that of *C. sinensis* (Figure 1C). Al treatment apparently increased the ratio of root to shoot of both *C. sinensis* and *C. grandis* (Figure 1F).

### 2.2. Effects of Al Treatment on Al Content and Root Lignin Content in Citrus

**Figure 2:**
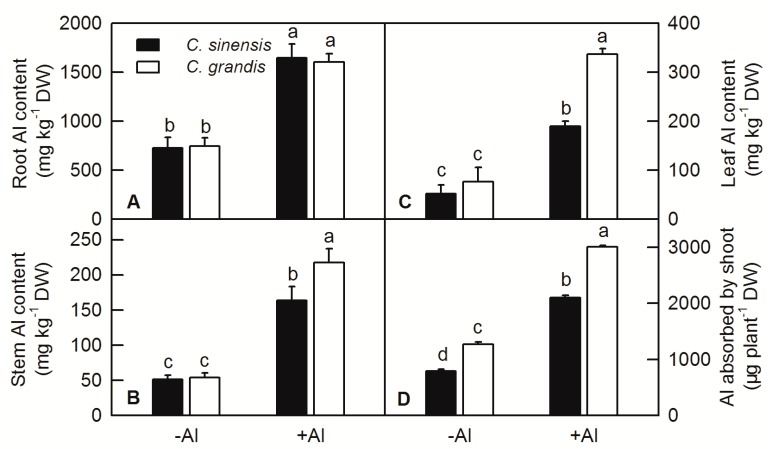
Effects of Al toxicity on Al content in root (**A**), stem (**B**), leaf (**C**) and Al absorbed by shoot (**D**) of *C. sinensis* and *C. grandis*. Bars represent means ± SD (*n* = 4). Different letters above the bars indicate a significant difference at *p* < 0.05.

Al treatment significantly increased the content of Al in the root, stem and leaf of *C. sinensis* and *C. grandis*, especially in the root. Under Al treatment, there is no difference of Al content between roots of *C. sinensis* and *C. grandis*, while the content of Al in the shoot and leaf of *C. grandis* is significantly higher than that in *C. sinensis*, indicating that *C. grandis* accumulated higher Al than *C. sinensis* under the same treatment conditions (Figure 2A–C). For the whole plant Al uptake, the total Al absorbed by a single seedling of *C. grandis* (based on the weight of stem and leaf) was significantly higher than that of *C. sinensis* (Figure 2D).

**Figure 3:**
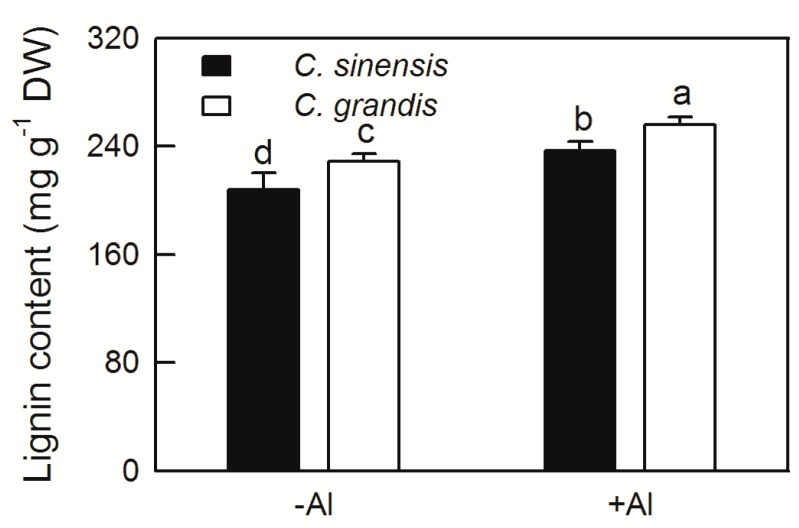
Effect of Al treatment on lignin content in roots of *C. sinensis* and *C. grandis*. Bars represent means ± SD (*n* = 4). Different letters above the bars indicate a significant difference at *p* < 0.05.

The increased content of plant lignin will affect cell wall elasticity and the elongation of developing cells. In this study, the lignin content of *C. sinensis* and *C. grandis* roots were significantly increased in various degrees by Al treatment. Furthermore, the lignin content of *C. grandis* roots was higher than that of *C. sinensis* at both −Al and +Al conditions (Figure 3).

### 2.3. Expression of ALS3 and CAD Genes in Citrus under Al Treatment

**Figure 4:**
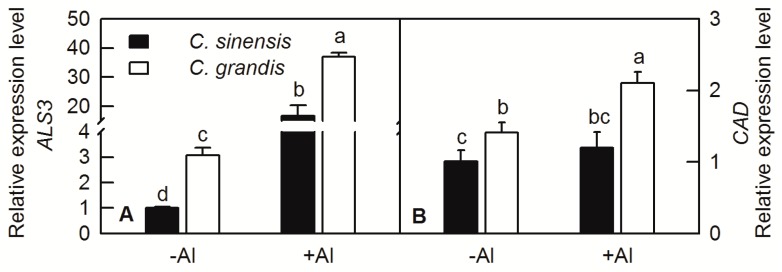
Relative expression levels of *ALS3* (**A**) and *CAD* (**B**) in *C. grandis* and *C. sinensis* under Al treatment. Bars represent means ± SD (*n* = 4). Different letters above the bars indicate a significant difference at *p* < 0.05.

The total RNAs were extracted from the roots of *C. sinensis* and *C. grandis* and relatively expression levels of *ALS3* and *CAD* under Al treatment were measured by RT-qPCR. The results showed that the expression levels of *ALS3* and *CAD* in *C. sinensis* and *C. grandis* were both increased under Al treatment (Figure 4).

### 2.4. Yeast Mating and Screening of Positive Colony

The recombinant pGADT7 plasmid containing candidate interacting protein genes were extracted from colonies after verified by colony PCR. The recombinant plasmids were sequenced and blasted against the citrus genome. Finally, 10 positive clones were identified as candidate interacting proteins of ALS3 and named as C51 (glutathione *S*-transferase, GST), C55 (vacuolar-type proton ATPase, V-ATPase)), C131 (aquaporin PIP2, PIP2), A58 (uncharacterized protein), A62 (fasciclin-like arabinogalactan protein 17, FAP17), A69 (auxin efflux carrier component 2, AECC2), A97 (ubiquitin carboxyl-terminal hydrolase 13, UCT13), A102 (uncharacterized protein1, UP1), A492 (putative dicyanin blue copper protein, DCBC) and A615 (uncharacterized protein 2, UP2). Nine positive clone colonies were identified as candidate interacting proteins of CAD and named as C51, C55, C120 (ALS3), C125 (cytochrome P450 71A1, CP450), C131, A58, A97, A102 and A615. Here, we found that C51, C55, C131, A58, A97, A102 and A615 were primarily identified as the candidate proteins interacted with ALS3 and CAD simultaneously. According to the sequencing results and the blast analysis against the citrus genome, the protein information is shown in Table 1.

**Figure 5:**
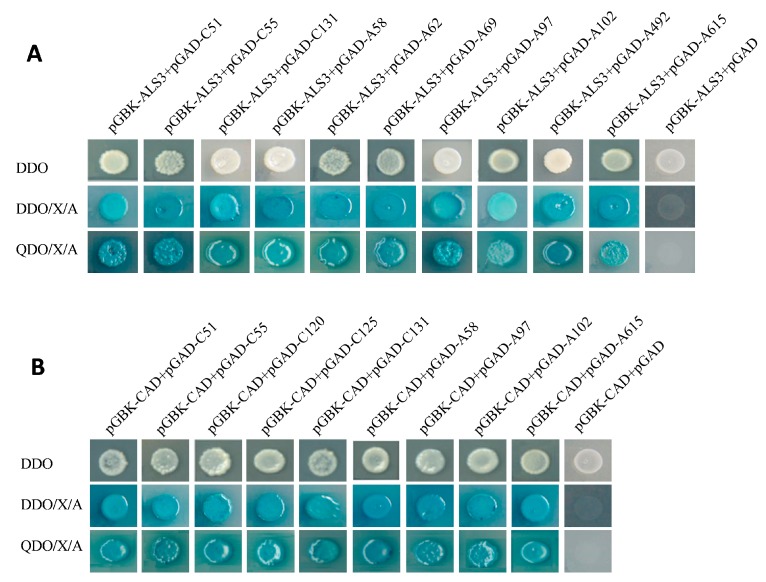
Candidate proteins interacted with ALS3 (**A**) and CAD (**B**) in yeast mating. Double-dropouts minimal media (DDO): single-dropouts (SD)/-Trp/-Leu; DDO/X/A: SD/-Trp/-Leu/X-a-Gal/ Aureobasidin A (AbA); quadruple-dropout/X/A (QDO/X/A): SD/-Trp/-Leu/-His/-Ade/Xa-Gal/AbA.

According to the gene sequence published in citrus genome, the full-length gene sequence of each candidate proteins were isolated, digested with restriction endonucleases and connected to the linear pGADT7. The recombinant pGADT7-candidate genes were transformed to Y187 strain and then mated with the bait strain containing pGBKT7-ALS3 and pGBKT7-CAD, respectively. The selected candidate colonies can grow normally on double-dropouts minimal media (DDO), DDO/X-a-Gal/Aureobasidin A (DDO/X/A), quadruple-dropout/X/A (QDO/X/A) plates, and can turn into blue on QDO/X/A plates (Figure 5A,B).

### 2.5. Verification of Candidate Proteins of ALS3 and CAD by BiFC

**Figure 6:**
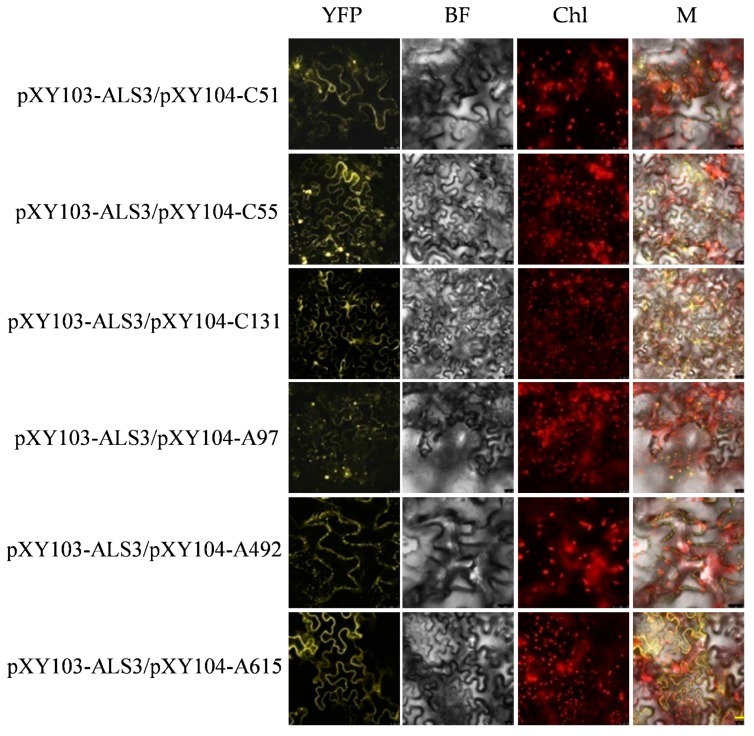
Fluorescence signal of ALS3 and its interacting proteins in tobacco mesophyll epidermal cells. YFP: yellow fluorescent protein; BF: bright field; Chl: autofluorescence of chloroplasts; M: merging of YFP, BF and Chl images. The value of scale bar with yellow color on the lower right of the figure is 25 μm.

**Figure 7:**
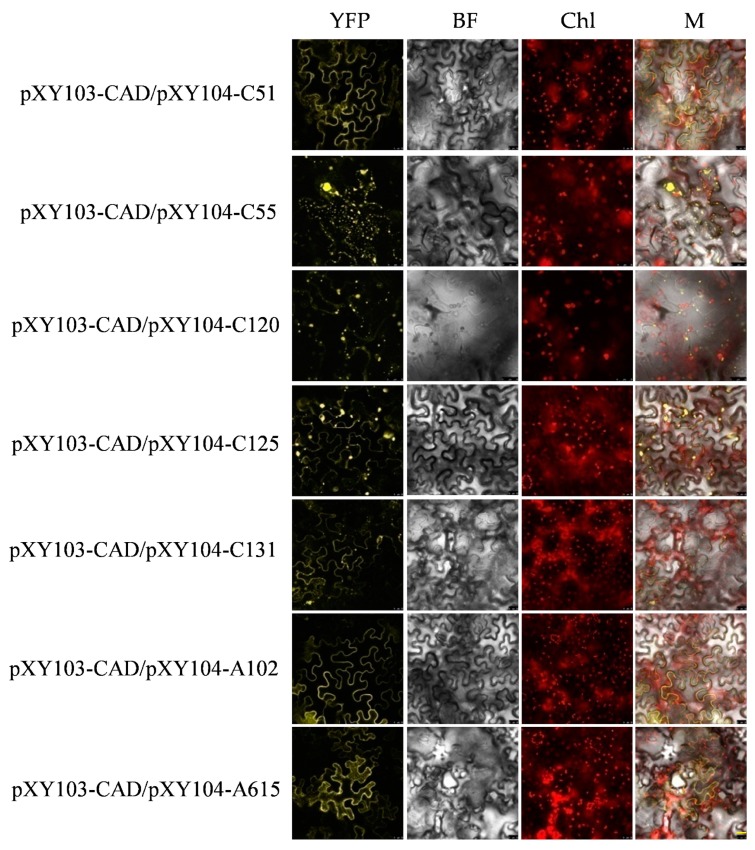
Fluorescence signal of CAD and its interacting proteins in tobacco mesophyll epidermal cells. YFP: yellow fluorescent protein; BF: bright field; Chl: autofluorescence of chloroplasts; M: merging of YFP, BF and Chl images. The value of scale bar with yellow color on the lower right of the figure is 25 μm.

The BiFC combined withY2H screening for interacting proteins can improve the reliability of the results and reduce the false positive rate. The recombinant plasmids of pXY103-ALS3-nYFP (N-terminal of yellow fluorescent protein), pXY103-CAD-nYFP, pXY104-candidate genes-cYFP (C-terminal of YFP) were transformed into Agrobacterium GV3101, respectively. Different combinations of positive Agrobacterium GV3101 containing recombinant pXY103 or pXY104 plasmids were infiltrated into tobacco leaves and monitored under confocal microscope (Leica TCS SP8, Weztlar, German). The combination of pXY103-ALS3-nYFP with pXY104-C51-cYFP, pXY104-C55-cYFP, pXY104-C131-cYFP, pXY104-A97-cYFP, pXY104-A492-cYFP and pXY104-A615-cYFP had a strong YFP fluorescent signal and most of them located in the cell membrane. The combination of pXY103-CAD-nYFP with pXY104-C51-cYFP, pXY104-C55-cYFP, pXY104-C120-cYFP, pXY104-C125-cYFP, pXY104-C131-cYFP, pXY104-A102-cYFP and pXY104-A615-cYFP had a strong fluorescent signal (Figure 6; Figure 7). Our BiFC results further confirmed the interaction relationship of ALS3 or CAD with their interacting proteins identified in Y2H.

### 2.6. RT-q PCR Analysis of ALS3 and CAD Interacting Protein Genes under Al Treatment

**Figure 8:**
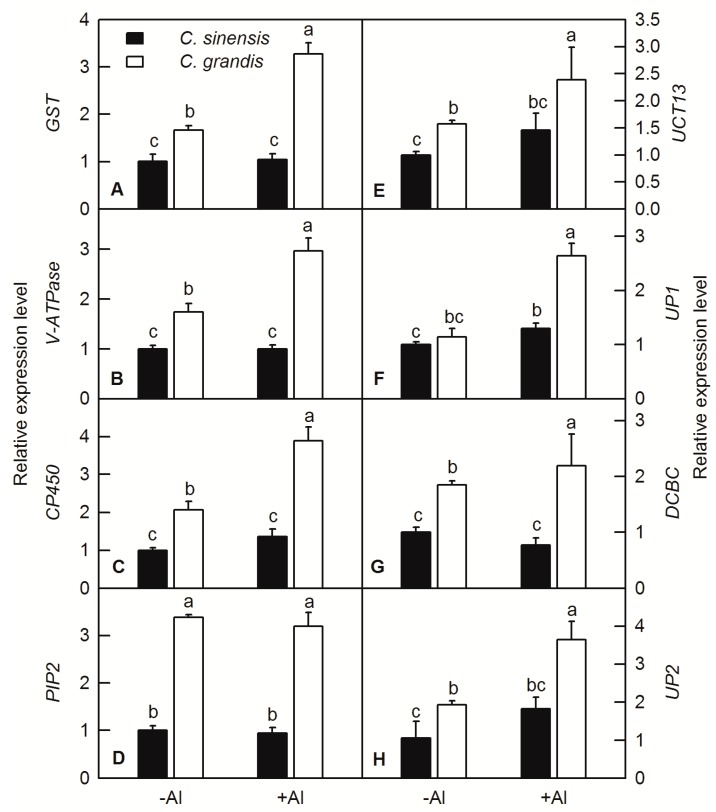
RT-qPCR analysis of the candidated interacting proteins of ALS3 and CAD in citrus roots. *GST*, glutathione *S*-transferase (**A**); *V-ATPase*, V-type proton ATPase (**B**); *CP450*, cytochrome P450 (**C**); *PIP2*, aquaporin PIP2 (**D**); *UCT13*, ubiquitin carboxyl-terminal hydrolase 13 (**E**); *UP1*, uncharacterized protein 1 (**F**); *DCBC*, putative dicyanin blue copper protein (**G**); *UP2*, uncharacterized protein 2 (**H**). Bars represent means ± SD (*n* = 4). Different letters above the bars indicate a significant difference at *p* < 0.05.

Gene special primer pairs of interacting proteins of ALS3 and CAD were designed and RT-qPCR was carried out. The results showed that Al treatment did not significantly affect the expression levels of the GST, V-ATPase, CP450, PIP2, UCT13, DCBC and UP2, but increased the expression levels UP2 in *C. sinensis* roots (Figure 8). Al treatment significantly increased the gene expression levels of the interacting proteins mentioned above in *C. grandis* roots, except for PIP2 (Figure 8).

## 3. Discussion

### 3.1. Effects of Al Treatment on the Growth and Al Distribution of C. sinensis and C. grandis

Al stress is one of the constraining factors that limited agricultural product yield and quality of crops cultivated on acidic soils [12]. Al-induced growth inhibition or crop yield reduction has been reported in a variety of plants, including rice [22], oil tea [23], *Arabidopsis* [24], sorghum [12], wheat [25,26], soybean [27], and citrus [13,20,21,28]. In the current study, beside the decreased DW of stem, shoot and whole plant (Figure 1), Al significantly increased the ratio of root/shoot DW in both *C. sinensis* and *C. grandis*, with more pronounced one in *C. grandis* than in *C. sinenesis* (Figure 1F). The increased value of root/shoot DW was also found in wheat, oil tea and citrus plants under Al treatment [20,23,25,28].

Root is the primary target of Al toxicity in plants [7]. The growth and functions of plant roots are inhibited by Al stress, resulting in a substantial reduction in shoot growth and eventually loss of crop yield. Janhunen et al. reported that Al caused nutrient imbalance, such as decreased calcium (Ca), magnesium (Mg), phosphorus (P), and increased nitrogen (N) and potassium (K) contents in shoots of Scots pine by affecting nutrient absorption of roots, although no visible change in root anatomy or in the number of mycorrhizas was observed [29]. The root DW was not changed by Al treatment in both *C. sinensis* and *C. grandis* (Figure 1A); however, the Al contents of root, stem, leaf, Al absorbed by shoot and lignin content in roots were significantly increased by Al (Figure 2 and Figure 3). Interestingly, the Al contents of stem, leaf and Al absorbed by shoot were higher in *C. grandis* than that in *C. sinensis* (Figure 3), which indicated that a higher portion of Al absorbed by roots was transported from roots to shoots in *C. grandis* than in *C. sinensis*. This result is consistent with higher expression level of *ALS3* in *C. grandis* roots than that in *C. sinensis* (Figure 4A). Lignin is the main structural material formed in the cell wall during secondary thickening. In general, the lignin content in cell wall of root tip cells will be increased in varying degrees by abiotic stress. Our recent study also showed that Al can trigger biosynthesis of lignin in *C. grandis* roots in presence of low B concentration in nutrient solution [13]. Al toxicity can induce the decrease of elasticity of cell walls by increasing the degree of lignification of cell walls and inhibited cell elongation [30]. Al significantly increased the lignin content of roots of *C. sinensis* and *C. grandis*, whereas that content of *C. grandis* roots was slightly higher than that of *C. sinensis* roots (Figure 3). This is in accordance with the higher expression level of CAD both in roots of *C. sinensis* and *C. grandis* under Al stress and in roots of *C. grandis* than that of *C. sinensis* (Figure 4B). The Al-induced up-regulation of CAD protein was also found in Al-sensitive sorghum cultivar SC566 [12]. Our recent published literature also found that CAD4 was significantly up-regulated by Al in *C. grandis* roots, which may be responsible for the higher content of lignin in *C. grandis* roots [13]. 

### 3.2. Interacting Proteins of ALS3 and CAD in C. sinensis and C. grandis

Combining the role of ALS3 in the re-distribution of Al in plant roots and shoots, and the role of root Al content in lignin biosynthesis via affecting the CAD activity, we conducted the Y2H and BiFC to identify the context and regulatory network of ALS3 and CAD in citrus. The gene expression levels of candidate proteins were also further testified by RT-qPCR.

Glutathione (GSH) plays an important role in plant detoxification and maintenance of redox status [31]. GST is one of the important enzymes in GSH metabolism. It catalyzes the binding of GSH to electrophilic substances and toxic substances. GSH can be transported into vacuoles through selective ATP-binding cassette (ABC) transporters to reduce the toxicity of harmful compounds such as heavy metals. Previous study showed that GST can regulate plant adaptability and tolerance, and play a key role in detoxification, stress resistance, defense, signal transduction and other functions in plants under adverse conditions [32]. Cicero et al. found that heterologous over-expression of *CsGSTU1* and *CsGSTU2* from citrus in tobacco can increase the tolerance of salinity and drought stress [33]. Over-expression of *GST/GPX* in tobacco under salt stress can increase the ability of tobacco to resist salt stress [34]. Our previous study reported that Al significantly increased the activity of GST in both roots and leaves of *C. grandis* and increase of S level in nutrition medium can further increase the activity of GST [28]. We found that GST interacted with both ALS3 and CAD, and the fluorescence signal was localized on the cell membrane and its adjacent area (Figure 6; Figure 7). RT-qPCR showed that the relative expression level of *GST* was significantly up-regulated by Al in roots of *C. grandis* (Figure 8A). According to these results, we speculated that GST may participate in the detoxification of Al cation in citrus roots via regulation of the GSH pool and detoxification of lipid hydroperoxides.

V-ATPase is widely distributed in the protoplast membrane of eukaryotic cells and the inner membrane of various organelles. It hydrolyzes ATP to release energy, produces a transmembrane electrochemical gradient, and promotes the transport of various ions and metabolites [35]. The lower pH in the cell wall could lead to physical loosening and inhibition of cell elongation [36]. V-ATPase controls cell elongation, secrete H^+^ ion, and acidify cell wall, which could facilitate lignin biosynthesis. Padmanaban et al. found that *AtVHA-c1* gene expressed in cotyledons, hypocotyls and root elongation regions under salt stress in *Arabidopsis* [37]. Our BiFC assay showed that the fluorescence signal of the combination of V-ATPase and ALS3 or CAD located in the cytoplasmic membrane and other endomembrane organelles (Figure 6; Figure 7), which was consistent with the results of Herman et al. and Sze et al. [38,39]. Analysis of ALS3 in *Arabidopsis* reveals that it does not possess an ATPase domain, which is required by ABC transporters for ATP hydrolysis to provide the necessary energy and expression of ALS3 in yeast without its cognate ATPase would result in non-functioning ALS3 [9]. Chen et al. reported that Al increased the activity of V-ATPase and uprising Mg level in nutrition medium could ameliorate Al toxicity by enhancing the phosphorylation levels of V-ATPase and its interaction with the 14-3-3b protein in broad bean [40]. Furthermore, inductions of V-ATPase were also observed in Al-treated roots of rice [22], soybean [41], tomato [42] and *C. grandis* [13]. Therefore, the induction of V-ATPase both in translational and transcriptional levels by Al in *C. grandis* roots and its interacting property with ALS3 and CAD implied its role in energizing ALS3 to export or import Al cations and facilitating lignin biosynthesis via proton pumping activity (Figure 8B) [13].

Phosphorylation is one of most important post-transcriptional modifications (PTMs) of functional protein in plants and plays a crucial biological regulation in plant growth and development [13,43]. Among the phosphorylation sites in amino acids, the most abundant phosphorylation events occur in serine or threonine residues in plants [43]. Serine-threonine protein kinase (STK) was demonstrated to be involved in physiological processes such as plant cell sugar metabolism, photosynthesis, cell growth and development [44]. Here, we reported that the UCT13, also named serine-threonine protein kinase, can interact with ALS3, but not CAD (Figure 6). The gene expression level of UCT13 was higher in *C. grandis* roots than in *C. sinensis* ones and induced by Al only in *C. grandis* roots (Figure 8E). This result implied that the normal function of ALS3 may also be involved in protein phosphorylation catalyzed by UCT13, and over-expression of both *ALS3* and *UCT13* in *C. grandis* roots may facilitate efflux of Al cation from roots to shoots.

Aquaporins are members of major intrinsic proteins (MIPs) that ubiquitously exist in the plasma membrane and vacuolar membrane in higher plants. Plasma membrane intrinsic protein (PIP) is one of the five subfamilies of MIP and could be divided into PIP1 and PIP2 subfamily [45]. Most studies have shown that aquaporins are highly specific and allow water molecules to pass without allowing other molecules and ions to pass through. However, it was later discovered that aquaporins can also transport other small molecules [46]. For example, PIPs family have been found to transport boron in rice and barley, which is a boric acid transport channel [47,48]. Two aquaporin family members identified in *Hydrangea* showed that *Hm*PALT1 can transport Al ions into the cytoplasm and *Hm*VALT can transport the Al ions into cell vacuole [49,50]. Our proteomic studies also found that B deficiency differentially regulated the protein abundance of PIPs in different citrus species [51,52]. We found that the PIP2 localized on the cell membrane and can simultaneously interact with ALS3 and CAD in citrus (Figure 6; Figure 7). RT-qPCR showed that the expression of *PIP2* in the roots of *C. grandis* was significantly higher than that of *C. sinensis* under any given Al treatment, but Al did not significantly increase the expression level of *PIP2* in the roots of *C. grandis* and *C. sinensis* (Figure 8D). Given the induction of ALS3 and its interactive relationship with PIP2, it is proposed that the activity of PIP2 is controlled by post-transcription modification as PIP2 is a kind of phosphoprotein and the phosphorylation role of another ALS3 interacting protein UCT13 (Figure 5; Figure 6) [53]. However, whether the clear function of PIP2 is a co-transport component of ALS3 in the transport of Al cation or just a channel involved in other small molecules transport needs a further functional analysis.

Cytochrome P450 (CP450) is one of the heme oxidoreductases and is involved in the biosynthesis of phenylpropanoid, flavonoid, coumarin, terpenoid, alkaloid, cyanogenic glucosides and the metabolism of lipid, xenobiotic compounds and detoxification of toxic substances in plants [54,55]. Available evidences showed that CP450 participated in hydroxylation, methylation and redox reactions in the phenylpropionic acid pathway and involved in the biosynthesis of lignin in plant cells [56,57]. The interaction between CP450 and CAD may further facilitate the biosynthesis of lignin. Our BiFC assay found that CP450 interacted with CAD, and the fluorescence signal mainly localized on the cytoplasmic membrane and some organelles (Figure 6; Figure 7), which is in line with previous reports [58]. Furthermore, RT-qPCR revealed that the expression level of CP450 significantly increased in the root of *C. grandis* under Al treatment (Figure 8C). The expression levels of *CP450* were also upregualted in rice under P deficiency [59], *C. sinensis* and *C. reticulata* under Mg deficiency [60,61], *Arabidopsis* under nitrogen deficiency [62] and *C. sinensis* under B deficiency [63]. Considering the over-expression levels of *CP450* and the interaction relationship between CP450 and CAD, we speculated that one of vital roles of CP450 can assist CAD to synthesize lignin in *C. grandis* roots.

Blue copper protein (DCBC) is a protein in the ferrous oxidation system and a member of the blue copper family. DCBC plays an important role in acid stabilization, redox homeostasis, and electron transport in plants, which exerts a great influence on plant growth and development [64,65,66]. Studies have shown that over-expression of *AtBCB* (a homologue of DCBC) in *Arabidopsis* could promote the synthesis of lignin, which enhanced the resistance of transgenic *Arabidopsis* to Al [67]. In our current study, the interaction of DCBC and ALS3 was detected in both Y2H and BiFC (Figure 6), but no interaction between DCBC and CAD was observed. RT-qPCR showed that there was no significant difference in the expression level of *DCBC* in *C. sinensis* roots under Al, but up-regulated that in *C. grandis* (Figure 8G). Considering the role of plant DCBC reported in previous studies, we suggest that DCBC participates in the transport efficiency of Al ions in cells or promotes the synthesis of lignin to achieve the ability to immobilize Al in citrus roots.

## 4. Materials and Methods 

### 4.1. Seedling Culture and Aluminum Treatments

The seeds of ‘Xuegan’ (*C. sinensis*) and ’Sour pummelo’ (*C. grandis*) were collected from Fujian Academy of Forestry Sciences, Fuzhou, China, and uniformed seeds were sown in a plastic tray. Six weeks after sprouting, uniform seedlings of *C. sinensis* and *C. grandis* were transplanted into 6 L pottery pots containing clean river sands (two seedings per pot), and then cultured under natural light at Fujian Agriculture and Forestry University, Fuzhou, China. The seedlings were irrigated every day with nutrient solution (about 500 mL per pot) after transplanting. The element concentrations in nutrient solution is as follows: 1 mM KNO_3_, 1 mM Ca(NO_3_)_2_, 0.1 mM KH_2_PO_4_, 0.5 mM MgSO_4_, 10 μM H_3_BO_3_, 2 μM MnCl_2_, 2 μM ZnSO_4_, 0.5 μM CuSO_4_, 0.065 μM (NH_4_)_6_Mo_7_O_24_, and 20 μM Fe-EDTA. After six weeks of transplanting, each pot was irrigated daily until dripping with the above nutrient solution containing 0 mM (control, -Al) or 1.0 mM (+Al) AlCl_3_·6H_2_O for 18 weeks. The pH of nutrient solution was adjusted to 4.08–4.15 with HCl or NaOH. After treatment, approximately 5-mm-long root tips from newly grown roots were excised and packaged into foil paper and immediately frozen in liquid nitrogen. The samples were stored at −80 °C until extraction.

### 4.2. Plant Dry Weight (DW), Al and Lignin Content in Roots of Citrus

Seedlings were separated into shoots and roots and loaded into different paper bags. The samples were oven-dried at 80 °C until constant weight was achieved. After the plant tissue was pulverized into fine powder, appropriately 0.2 g sample was digested with HNO_3_: HClO_4_ (5:1 *v/v*) and the Al content was determined by the colorimetric method [68]. The lignin content of roots was measured according to the method described by Morrison [69]. Briefly, about 100 mg root tissue was ground in 95% ethanol. After centrifugation at 1000 g for 5 min, the precipitation was washed three times with 95% ethanol. The solution of ethanol-hexane was used twice to wash the precipitation with 1:2 (*v/v*) later. After vacuum drying, 10 mg sample powder was soaked in glacial acetic acid containing 25% bromoacetic acid for 30 min at 70 °C. Then 0.9 mL 2 M NaOH, 5 mL glacial acetic acid and 0.1 mL 7.5 M hydroxylamine hydrochloride were added. After mixing, the solution was centrifuged at 1000 g for 5 min. The absorbance of supernatant was measured at 280 nm. The experiment was set up with four replicates.

### 4.3. RT-qPCR Analysis of ALS3 and CAD

Total RNA was extracted from about 0.5 g root samples using TRIzol reagent (Invitrogen, Carlsbad, CA, USA) and reversely transcribed into cDNA according to user manual of FastKing first strand cDNA synthesis kit (Tiangen, Beijing, China). Briefly, a total of 10 μL mixture contained 2 μL 5× reaction buffer, 2 μg total RNA, 1 μL RT enzyme mix, 2 μL oligo dT and adequate RNase-free water. There were four biological replicates per treatment. Gene specific primers were designed and accessed using Premier Primer 5.0 software (Premier Biosoft International, Palo Alto, CA, USA). The primers were synthesized by BGI (Beijing, China) and the sequence information of primers is shown in Table S1. RT-qPCR was performed according to the instructions of the iQ SYBR Green supermix quantification kit (Bio-Bad, Hercules, CA, USA). A total of 20 μL mixture contained 10 μL iQ SYBR green supermix, 10 μM forward primer, 10 μL reverse primer, 2 μL cDNA template and adequate nuclease-free water. The PCR procedure was listed as follow: 95 °C, 2 min; 40 cycles of 95 °C, 10 s, 60 °C, 30 s, 72 °C, 30 s. The relative gene expression level was calculated using the ddCt algorithm (2^−ΔΔct^), and the U6 gene (accession number: Ciclev10031363m) was used as the internal reference. The -Al sample was used as the control, the gene expression levels of which were set to 1. There were three technical replicates for each gene.

### 4.4. Y2H cDNA Library Construction and Screening of Interacting Proteins of ALS3 and CAD

Total RNAs were extracted from citrus roots and reversely transcribed into cDNA using the SMART technique. The construction of Y2H cDNA library was performed by the method described in Make Your Own “Mate & Plate” Library System (Clonetech Laboratories). Briefly, the full length of *ALS3* and *CAD* were cloned into a pGBKT7 DNA-BD cloning vector and transformed to the Y2HGold yeast strain (Clonetech Laboratories). After selection of single-dropouts (SD)/-Trp plate, 4 mL positive Y187 clone containing *ALS3* or *CAD* was mixed with 1 ml of Y2H cDNA library and incubate overnight after add 45 mL 2xYPDA, respectively. Then, 150 μL bacterial suspensions were spread on SD/-Trp/-Leu/X-a-Gal/AbA (DDO/X/A) 150-mm selective media (about 30 plates each) and incubated at 30 °C for 5–7 days. Later, all the blue colonies that grew on DDO/X/A were picked onto higher stringent SD/-Trp/-Leu/-His/-Ade/Xa-Gal/AbA (QDO/X/A) medium agar plates using a flat sterile toothpick. After incubation at 30 °C for 5–7 days, the blue single colonies were selected for colony PCR to identify positive colonies. The positive clones verified by gene special PCR were extracted and sequenced. Finally, the sequencing results were blasted against citrus genome. The full length of genes of identified candidate proteins were isolated and cloned into pGAD-T7, transformed into yeast Y187 strain and selected by plating on SD/-Leu at 30 °C for 3–5 days. The single positive Y187 colony of each candidate protein was mated with the bait colony containing pGBKT7-ALS3 and pGBKT7-CAD, respectively. The mixed suspension was spotted on the DDO, DDO/X/A and QDO/X/A 150-mm agar plates, respectively, and cultured at 30 °C for 3–5 days. The yeast mating was conducted in three replicates.

### 4.5. Verification of Candidate Interacting Proteins of ALS3 and CAD by BiFC Assay

Full-length gene sequences of candidate interacting proteins were isolated and recombined with the pXY104 vector infused with the C-terminus of yellow fluorescent protein (YFP). The full-length of *ALS3* and *CAD* were recombined with the pXY103 vector with the N-terminus of YFP. The recombinant vector was transferred into Agrobacterium GV3101 and cultured in LB/rifampicin (Rif)/spectinomycin (Spec) plates at 28 °C until the apparent clone appeared. After incubation at 28 °C for 3 days, single colonies were picked up to 5 mL of LB liquid medium (containing 50 mg·mL^−1^ Rif, 100 mg·mL^−1^ spectinomycin) and cultured at 28 °C and 200 rpm until OD = 0.4–0.6. The Agrobacterium was collected by centrifuging at 3500 r/min for 10 min. The pellet was washed with 15 mL 10 mM MgCl_2_ solution. The pellet was added a mixture of an equal volume of 10 mM magnesium chloride and 200 μmol·L^−1^ acetosyringone solution and re-suspended evenly. The Agrobacterium containing pXY104 infused with the candidate gene were mixed with the equal volume of Agrobacterium containing pXY103-ALS3 and pXY103-CAD, respectively, and left at room temperature for 2 h. The bacterial solution was aspirated with a 2 mL sterile syringe and slowly infiltrated tobacco leaves. After two days of infiltration, the infected leaves were sliced and placed under laser con-focal microscope to observe the signal of YFP fluorescence. The wavelength of excitation light is 488 nm. The chloroplast fluorescence acquisition wavelength is 650–750 nm, and the scale is 25 μm. The BiFC assay was conducted in three replicates.

### 4.6. RT-qPCR Analysis the Interacting Proteins of ALS3 and CAD

Total RNAs were isolated from different samples and reversely transcribed into cDNA according to user manual of FastKing first strand cDNA synthesis kit (Tiangen, Beijing, China). Gene special primer pairs were designed using Premier Primer 5.0 software (Premier Biosoft International, Palo Alto, CA, USA) according to the their sequences deposited in citrus genome (Table S1). RT-qPCR was performed by the method described above. There were four replicates for RT-qPCR.

### 4.7. Experimental Design and Statistical Analysis

There were 20 pots for each treatment in a completely randomized design. Experiments were conducted with fouror 10 replicates (one plant per replicate). There were 10 replicates for plant biomass, four replicates for RT-qPCR, Al and lignin contents measurement, respectively. Results were displayed as means ± SD for *n* = 4 or 10. Means were separated by the least significant difference method (LSD) at *p* < 0.05.

## 5. Conclusions

Al treatment significantly decreased the DW of stem, shoot and whole plant of both *C. sinensis* and *C. grandis*, but did not change that of root. Al significantly decreased leaf DW of *C. grandis*, increased the ratio of root to shoot and the content of lignin in roots of both species. The higher content of Al in leaves and stems, and lignin in roots of *C. grandis* than that of *C. sinensis* might be due to the over-expression of ALS3 and CAD in roots of *C. grandis*, respectively. By using Y2H and BiFC techniques, we obtained the results that GST, V-ATPase, PIP2, UCT13, DCBC and UP2 were interacted with ALS3 and GST, V-ATPase, ALS3, CP450, PIP2, UP1 and UP2 were interacted with CAD. Annotation analysis revealed that these proteins were involved in detoxification, cellular transport, post-transcriptional modification, and oxidation-reduction homeostasis or lignin biosynthesis. RT-qPCR analysis further revealed that the higher gene expression levels of most of these interacting proteins in *C. grandis* roots than that in *C. sinensis* ones were consistent with the higher contents of lignin in *C. grandis* roots and Al absorbed by *C. grandis*. This study identified some key interacting components of Al responsive proteins ALS3 and CAD (Figure S1), which could further help us to understand the molecular mechanism of Al tolerance in citrus plants and provide new information to the selection and breeding of tolerant cultivars, which are cultivated in acidic areas.

## Figures and Tables

**Table 1:** Analysis of the inserted sequences from screening of cDNA library.

Y2H Code	Gene ID Number	Gene Annotation
C51	Cs5g32800.1	Glutathione *S*-transferase, GST
C55	Cs3g25710.1	Vacuolar-type proton ATPase, V-ATPase
C120	Cs6g03670.1	Aluminum Sensitive 3, ALS3
C125	Cs1g17390.1	Cytochrome P450 71A1, CP450
C131	Cs8g16640.1	Aquaporin PIP2, PIP2
A58	orange1.1t03320	Uncharacterized protein
A62	Cs6g13410.1	Fasciclin-like arabinogalactan protein 17, FAP17
A69	Cs4g04340.1	Auxin efflux carrier component 2, AECC2
A97	orange1.1t04179.1	Ubiquitin carboxyl-terminal hydrolase 13, UCT13
A102	Cs8g03780.1	Uncharacterized protein1, UP1
A492	Cs4g18120.1	Putative dicyanin blue copper protein, DCBC
A615	Cs7g02860.1	Uncharacterized protein2, UP2

## Supplementary Materials

The following are available online at https://www.mdpi.com/1422-0067/20/19/4846/s1, Figure S1: Graphical diagram of the functions of ALS3, CAD and their interaction proteins. Table S1: RT-qPCR primers for ALS3 and CAD and their interacting proteins.
