# Supplementary material for: Analysis of Interacting Proteins of Aluminum Toxicity Response Factor ALS3 and CAD in Citrus
Source: Int J Mol Sci. 2019 Sep 29;20(19):4846. doi: 10.3390/ijms20194846 (PMC6801426; doi:10.3390/ijms20194846)
Supplement: Supplementary file 1 [file ijms-20-04846-s001.zip › Figure S1.pdf]

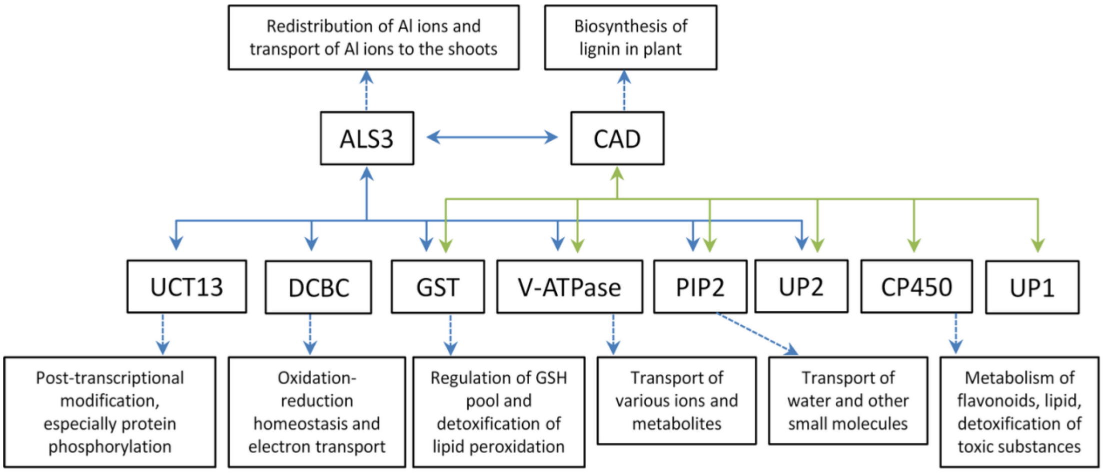

Figure S1. Graphical diagram of the functions of ALS3, CAD and their interaction proteins. Blue and green solid arrows indicate this protein interacts with ALS3 or CAD, respectively. Dotted arrow indicates the function of this protein.
